# Trait agreeableness moderates the relationship between induced state agreeableness and feeling states

**DOI:** 10.3389/fpsyg.2025.1646442

**Published:** 2026-01-14

**Authors:** Sointu Leikas

**Affiliations:** Helsinki Institute for Social Sciences and Humanities, University of Helsinki, Helsinki, Finland

**Keywords:** affect, daily process design, intervention - behavioral, personality, states, traits

## Abstract

The idea that counterdispositional behavior is depleting, stressful, or less rewarding than trait-consistent behavior has received much attention in personality science in recent years. In an experience sampling study with a within-person intervention design (*N* = 74), participants first went through a baseline ESM protocol and were then prompted to behave in an agreeable and conscientious way for 4 days each. Trait Agreeableness moderated the relationship between agreeable behavior and momentary feelings; the higher the participant's trait Agreeableness, the better and less stressed they felt after behaving agreeably. In contrast, trait Conscientiousness did not moderate conscientious behavior-outcome relations. The results were in line with previous studies on counterdispositional Conscientiousness and suggested that those with higher trait Agreeableness may find it easier or more rewarding to act agreeably than those with lower trait Agreeableness.

## 1 Introduction

After experience sampling methods became widespread in personality science (Fleeson, 2001; Fleeson and Gallagher, 2009; Hamaker and Wichers, 2017), studying the variability of personality trait-relevant behavior or personality states (i.e., states or momentary behavior with the same content as personality traits, such as momentary sociability or agreeableness) in everyday life became much easier. As a result, the question of the possible consequences of counterdispositional behavior—behavior that is not in line with one's stable personality dispositions—has gained considerable interest (e.g., Fleeson et al., 2002; Fleeson and Wilt, 2010; Jacques-Hamilton et al., 2019; Kuijpers et al., 2024; Leikas et al., 2021; McNiel and Fleeson, 2006; Wilt et al., 2023; Zelenski et al., 2012). This is no surprise, as questions about whether personality affects the way we feel after behaving in, say, an agreeable or confident way, have great theoretical and practical relevance.

Many studies have been conducted on these questions in the domain of naturally occurring relationships between personality-related behavior (or personality states) and feeling states (e.g., Ching et al., 2014; Fleeson et al., 2002; Kritzler et al., 2020; Leikas et al., 2021), and a substantial amount of literature has dealt with the effects of experimentally or quasi-experimentally manipulated behavior on wellbeing indicators (e.g., Jacques-Hamilton et al., 2019; Margolis and Lyubomirsky, 2020; McNiel et al., 2010; Zelenski et al., 2012). However, the latter literature has largely focused on Extraversion and extraverted behavior, and less is known about the dynamics between activation of other trait domains and wellbeing. This study addresses this issue by investigating agreeable and conscientious behaviors and feelings in a within-subject experience sampling study in an intervention framework.

Questions about the role of personality traits in the dynamics between behavior, affect, and wellbeing are highly relevant to personality theory and to all-but-resolved questions about the ontology of personality traits (Block, 1995; Fleeson and Jayawickreme, 2015; Franić et al., 2014; Mõttus, 2016). If personality traits indeed moderate the relationship between trait-related behavior and outcomes, this suggests that not only do people behave in line with their personalities more often than not (Fleeson and Gallagher, 2009), but that traits also help (or hinder) us to behave in a certain way. For instance, if people with low trait Conscientiousness turn out to be more tired or stressed after behaving in a conscientious way than people with high trait Conscientiousness, this would suggest that higher levels of trait Conscientiousness would make it less resource-consuming and/or less stressful to act conscientiously, and lower levels of trait Conscientiousness would, in turn, make it more difficult.

Of course, results such as those hypothesized above would not provide conclusive evidence about the postulated dynamics, given the multidetermined nature of behavior and affective experiences, and because a strictly controlled, randomized experiment on this topic that would still capture the natural course of everyday life would be nearly impossible to implement. However, such results would show that one's personality trait level is connected to the ease or difficulty of trait-related behavior. Consequently, such results would give some substance to the notion of traits as dispositions or behavioral tendencies. For instance, perhaps individuals with high trait Conscientiousness behave in a conscientious way more often than individuals with low trait Conscientiousness (e.g., Fleeson and Gallagher, 2009) because it is easier for the former group? Conversely, if personality traits do not moderate the behavior-affect or behavior-wellbeing relationships, this would suggest that we probably need to understand traits in ways unrelated to ease or difficulty or trait-consistent behavior. Thus, studying the potential moderating effect of personality traits on behavior-outcome relations advances trait theory.

The topic of counterdispositional behavior also carries considerable empirical interest—finding out whether engaging in behavior that is inevitably needed in everyday life makes one, for instance, more tired than it makes most others relevant to everyday life. Somewhat highly agreeable behavior is often required to get along with colleagues or neighbors, and for people working in customer service, even very high levels of agreeableness can be necessary on occasion. The same applies to the necessity of at least some level of conscientious behavior in work life and studies. If a behavior that is an integral part of everyday life is more stressful or tiring for some, it would be useful to better understand this dynamic. This would make it possible to develop a means to nurture social relationships and fulfill duties without exhaustion and stress.

The idea of trait-consistent behavior as somehow easier is intuitively compelling. Theories about the self and authenticity have typically suggested that behaving in ways consistent with one's true self results in higher wellbeing and feelings of authenticity (Goldman and Kernis, 2002; Swann and Read, 1981). While this may be true with regard to complex inner dynamics (Rogers, 1961; Winnicott, 1965), a large proportion of counterdispositional behavior research based on major personality trait models has suggested the opposite. Within the Big Five/Five-Factor/HEXACO frameworks, many studies have shown that people feel more positive and less negative affect and feel more authentic and less stressed and tired when behaving in more extraverted, emotionally stable, agreeable, open, and conscientious ways (Fleeson and Wilt, 2010; Ching et al., 2014; Kritzler et al., 2020; Leikas and Ilmarinen, 2017; Wilt et al., 2012), regardless of their personality trait levels (Fleeson and Wilt, 2010; Kuijpers et al., 2024; Leikas et al., 2021). However, some studies have suggested that counterdispositional behavior may have negative affective consequences. (Côté and Moskowitz 1998) found a counterdispositional cost effect for the trait Agreeableness on affect, and (Kuijpers et al. 2022) found a general counterdispositional cost effect by analyzing all trait domains together (using ESM-based averaged behaviors instead of personality traits as the indicator of dispositional/habitual behavior).

The above-listed studies have analyzed naturally occurring concurrent or lagged relations between behaviors and feeling states and thus cannot be used to conclude whether desirable behavioral states increase positive feelings or vice versa, or whether these relationships are due to a third variable causing both (for instance, it may be that when people are having a generally positive moment, they are likely to both experience elevated moods *and* engage in desirable behaviors). Nevertheless, according to the current body of evidence, the relationships between trait-relevant behavior and feeling states in natural life seem to be largely unmoderated by personality traits.

Several studies have also investigated how experimental or quasi-experimental interventions targeting trait-relevant behaviors affect feeling states. These studies have mostly focused on Extraversion, and they have typically shown that experimentally induced extraverted behavior causes more positive feeling states regardless of the levels of participants' trait Extraversion (Fleeson et al., 2002; Margolis and Lyubomirsky, 2020; Zelenski et al., 2012, but see Jacques-Hamilton et al., 2019, for a single result of trait Extraversion moderating the effect, and Fritz et al., 2023; for a null effect on mood compared to a control group). One study also manipulated neuroticism (low emotional stability) and found that induced neuroticism led to lower mood but did not test moderation by trait Emotional Stability/Neuroticism (McNiel et al., 2010).

A handful of studies have also investigated prosocial interventions—prompting people to commit prosocial acts [see (Byrne et al. 2023) for a recent meta-analysis of the health effects of such interventions]. These studies can be seen as relevant for the personality domain trait Agreeableness, and they have shown that engaging in prosocial acts is related to increased wellbeing (Layous et al., 2017). However, these studies have rarely investigated trait Agreeableness as a moderator.

As discussed earlier, most existing evidence suggests that the relationships between momentary behaviors and momentary affective experience and other wellbeing indicators are not moderated by personality traits, supporting the idea that behaving according to one's personality is not easier or more pleasant, and behaving against one's personality is not more difficult or more unpleasant. Nevertheless, the existing literature is far from conclusive. Almost all personality-relevant studies using experimental or intervention approaches have focused on extraverted behavior; therefore, little is known about the effects of induced behavior on other trait domains. In addition, given the strong and robust link between sociability and happiness and positive affect, it may be that Extraversion is not the domain in which the potential counterdispositional effects would be most readily found, given the strength of the main effect of sociability on positive outcomes.

There may also be methodological caveats in studying counterdispositional behavior using observational (i.e., non-intervention) designs. While observational ESM designs are highly valuable in that they provide a window to the natural course of people's everyday lives, an issue in investigating counterdispositional behavior with such designs is that, in everyday life, most people exhibit a somewhat large variability in behavior related to all major trait domains (Fleeson, 2001; Fleeson and Gallagher, 2009). Therefore, it may be that individuals exhibit a relatively wide range of behavior along a given behavioral dimension without experiencing it as “countering” one's personality or typical behavior in a subjectively meaningful way. In addition, different people may have different criteria for judging their behavior as more or less extraverted, agreeable, and so on, which may introduce unaccounted bias into the between-person estimates. Finally, laboratory studies of induced behavior (Fleeson et al., 2002; McNiel et al., 2010; Zelenski et al., 2012) are the only designs that can offer information about causal processes and are invaluable; however, they can only investigate situations removed from the natural flow of life (Jacques-Hamilton et al., 2019).

ESM intervention designs, while not without their own caveats, can provide helpful information that overcomes, to an extent, the limitations of the observational and laboratory designs discussed above. First, when a given behavior is successfully induced via a prompt, we know that there is a shift in that behavior when compared to the natural course of participants' lives. Thus, after successful induction, participants' behavior on the given dimension will very likely be higher than it otherwise would have been for each participant (at that moment). Second, induced behavior is likely to be less susceptible to subjective criteria when the level of behavior is low, average, or high. Finally, ESM-based intervention studies are implemented over the course of natural life, thus overcoming the ecological validity limitations of laboratory studies. In sum, ESM intervention studies offer a way of studying whether traits moderate the relationship between somewhat objective increases in the levels of a given behavior and wellbeing indicators.

As noted, several natural life ESM interventions have been conducted with regard to Extraversion, most showing a positive effect of induced extraverted behavior, but usually no moderation by trait Extraversion (Margolis and Lyubomirsky, 2020; van Allen et al., 2021; but see (Jacques-Hamilton et al. 2019), for an exception). In the present study, this framework is extended to Agreeableness and Conscientiousness interventions. These trait domains are highly relevant for everyday life, as it seems reasonable to assume that at least somewhat agreeable behavior is typically needed in most human interactions and at least somewhat conscientious behavior at work, in studies, and in other duties of life. Consequently, both agreeable and conscientious behaviors may sometimes need to be enacted against people's will or motivation, or even when they lack adequate psychological resources to do so. For instance, university students—a commonly studied population in the relevant literature—often need to remain agreeable toward fellow students when working on group assignments, even when facing disagreement, and to prepare for an exam even after a poor night. Thus, these trait domains might be likely to show moderation by traits—perhaps more likely than the Extraversion domain, which is strongly and robustly correlated with wellbeing and positive affect, both at the trait and state levels.

This quasi-experimental intervention study investigates the relationship between induced agreeable and conscientious behavior and affect, stress, and fatigue, and the moderating effects of trait Agreeableness and Conscientiousness on these relationships. It should be noted here that in studies investigating counterdispositional behavior in ESM designs, there are currently two ways to conceptualize the dispositional tendency to behave in a certain way: using personality measured with traditional trait questionnaires (Jacques-Hamilton et al., 2019) or using averaged behavioral summaries derived from the ESM procedure itself (Kuijpers et al., 2022). Both approaches have advantages. In this study, traditional trait measures were used because the baseline ESM phase was short (only 16 measurement occasions) to reduce participant burden. Therefore, using baseline behavior/personality state reports as a baseline was not deemed sufficiently reliable to tap stable dispositional tendencies.

This study was conducted without preregistration. Data, analysis code, details of power calculations, and supplementary images and tables are available in an Online Appendix S1.

## 2 Materials and methods

### 2.1 Participants

Participants were recruited via e-mail invitations to student mailing lists of four Finnish universities. The invitations invited students to participate in a study on daily behavior that would last 3 weeks, 4 days per week. The invitation further explained that the first week would be a baseline week during which they would answer brief questionnaires about their behavior and mood, and that during the latter 2 weeks, they would be prompted in the morning to engage in a certain type of behavior, followed by behavior and mood questionnaires. In addition, they completed a background questionnaire consisting of personality trait measures and demographic questions. As compensation, participants received a gift card worth 20 euros from a major department store chain.

Seventy-six students agreed and completed the background questionnaire, but two did not participate in the ESM phase. Thus, the final sample size was 74 participants (58 women and 16 men). They were, on average, 26.8 years old (*SD* = 6.6 years, range = 19–54 years). As for their main occupation, 62 (83.8) % reported being full-time students, 8 (10.8%) reported working full-time, and 4 (5.4%) reported “other.” Regarding relationship situation, 29 participants (39.2%) reported being single, 22 (29.7%) dating, 16 (21.6%) cohabiting, 6 (8.1%) married, and one (1.4%) divorced. The data were collected in May–June 2017. According to the guidelines of the hosting institution and country-level science ethics guidelines (TENK, 2019), this study did not require an ethics approval statement. Therefore, none was sought. Participants provided informed consent, and their ESM responses were linked to each other and to the background questionnaire using a non-identifying code word (e.g., pine, coffee), which ensured that responses could not be linked to participants' identities in any way.

### 2.2 Intervention prompts

The prompts were constructed by the author for the purposes of the present study because, at the time of data collection, no standard state Agreeableness or state Conscientiousness manipulation existed. It was also necessary to focus on certain aspects of these broad traits instead of attempting to manipulate all trait-relevant states. For Agreeableness, it was decided to focus on kindness, empathy, and unselfishness, and for Conscientiousness, on dutifulness and deliberation, because for most people, daily life naturally offers opportunities to act along the continuum of these state dimensions, arguably more so than for other Agreeableness facets such as modesty or straightforwardness, or for other Conscientiousness facets of competence or achievement striving. It was also deemed important to ensure that participants would actually do something along the lines of the intervention, so the prompts were constructed to induce a concrete act [coincidentally, the prompts ended up resembling the behavioral “challenges” later created in the personality trait change literature for Agreeableness and Conscientiousness interventions; see (Hudson et al. 2019)]. The final prompts were “Today, when you get a chance, do something kind, compassionate, or unselfish” (agreeableness) and “Today, when you get a chance, do something responsible and careful” (conscientiousness).

### 2.3 Procedure

Participants completed the background questionnaire online 1–2 weeks prior to the baseline week ESM phase. In the baseline week, from Monday to Thursday, participants received four text messages each day (at 11 a.m., 3 p.m., 6 p.m., and 9 p.m.) containing the link to the ESM questionnaire. The second week was the agreeableness prompt week. During this week, from Monday to Thursday, participants received a text message each morning at 9 a.m., saying, “This is a reminder from the daily behavior study. Today, when you get a chance, do something kind, compassionate, or unselfish.” Then, they received four text messages at 1 p.m., 4 p.m., 7 p.m., and 9 p.m., each containing a link to the ESM questionnaire. The questionnaire first asked, “This morning, you were asked to do something kind, compassionate, or unselfish when you got the chance. Have you done that already?” followed by a yes/no question and the affect and behavior questions outlined below. Furthermore, if participants reported having already conducted the kind act, they were asked, “How much time has passed since your kind act?” and responded on a 4-point scale with options of 1 (*less than an hour*), 2 (*1–2 h*), 3 (*2–3 h*), and 4 (*more than 3 h*). After a participant responded “yes” to the yes/no question about completing the prompt, all further responses from that day were classified as “after the prompt” responses.

The third week was the conscientiousness-prompt week. Its procedure was identical to that of the agreeableness week, except that the morning message read, “This is a reminder from the daily behavior study. Today, when you get a chance, do something responsible and thorough,” and the questionnaire opening caption was “This morning, you were asked to do something responsible and thorough when you got the chance. Have you done that already?” All the data were collected in Finnish.

### 2.4 Measures

#### 2.4.1 Momentary behavior

Momentary behavior was measured by asking participants, in each ESM questionnaire, to “evaluate their behavior during the last hour. Have you been…” followed by personality trait-based items. The items focused on the same aspects as the intervention and were loosely based on the Big Five Inventory (Goldberg, 1999). Momentary agreeableness was measured using *friendly, compassionate, unselfish*, and *rude* (reversed) items, whereas momentary conscientiousness was measured using *responsible, productive*, and *sloppy* (reversed) items. These items were chosen on empirical grounds: (a) on the basis of previous experience sampling studies measuring state agreeableness and conscientiousness (Fleeson, 2001; Leikas et al., 2021) and (b) to cover the same trait content as the intervention prompts.

Extraverted and emotionally stable behaviors were also measured using single items, *sociable* and *insecure* (reversed), respectively, but these ratings are not used in this study. All ratings were made on a scale ranging from 1 (*not at all*) to 5 (*very much*). Within- and between-person omega reliabilities for momentary behaviors, computed using the function omegaSEM from the multilevelTools package (Wiley, 2025), are presented in Table 1 for the baseline, agreeableness, and conscientiousness weeks, respectively.

**Table 1:** Omega reliabilities for the ESM measures.

**ESM measure**	**Baseline week**	**Agree. week (after)**	**Consc. week (after)**
Positive affect	0.73/0.96	0.67/0.99	0.75/0.94
Negative affect	0.64/0.92	0.66/0.91	0.66/0.85
Agreeableness	0.69/0.92	0.62/0.93	0.64/0.94
Conscientiousness	0.65/0.80	0.71/0.80	0.69/0.86

Within/between-person reliabilities are presented for each multi-indicator measure. Omega reliabilities were computed using the omegaSEM function from the multilevelTools package (Wiley, 2025).

#### 2.4.2 Momentary feelings

Momentary feelings [positive (PA) and negative affect (NA), stress, and fatigue] were measured by asking participants to “evaluate their feelings and tiredness at the moment.” “Are you…?” This was followed by *happy* (PA), *content* (PA), *dejected* (NA), *irritable* (NA), *anxious* (NA)*, stressed out* (stress), and *tired* (fatigue) items, all rated on a scale of 1 (*not at all*) to 5 (*very much*). The within- and between-person omega reliabilities for positive and negative affect and stress are presented in Table 1. *Tired* was used as a single item to measure momentary fatigue.

#### 2.4.3 Personality traits

Personality traits (Agreeableness and Conscientiousness) were measured with the NEO-FFI-R (McCrae and Costa, 2004), in which each trait was measured with 12 self-report items. NEO-FFI-R is a shortened version of the NEO-PI-R measure (Costa and McCrae, 2008), which has been translated into Finnish and validated in Finland (Lönnqvist and Tuulio-Henriksson, 2008). Example items for the trait Agreeableness include *I usually try to be thoughtful and considerate*, and *Many people think of me as somewhat cold and distant* (reversed). Example items for the trait Conscientiousness include *I try to perform all the tasks assigned to me conscientiously* and *I waste a lot of time before settling down to work* (reversed). The items are rated on a scale of 1 (*fully disagree*) to 5 (*fully agree*). Cronbach's alpha (Omega total) reliabilities for the traits were adequate: 0.73 (0.70) for Agreeableness and 0.86 (0.86) for Conscientiousness.

### 2.5 Analytical strategy

The original analysis plan was to compare “before” and “after” conditions from the two intervention weeks to baseline and to compare before and after conditions to each other within each week, including the moderating effect of the corresponding personality trait. Unfortunately, initial examination of the data revealed that each day, a large number of participants performed the agreeable or conscientious act before the first questionnaire prompt of the day on all 4 days and thus did not provide any “before” ratings. In the agreeableness week, 42 out of 74 participants (56.8%) provided one or more before ratings, whereas 72 out of 74 provided after ratings. In the conscientiousness week, only 31 participants (41.9%) provided one or more before ratings (whereas 70 participants provided after ratings). Given the large number of missing values in the before condition, multiple imputation was not deemed reliable, and dropping level 2 *N* to 31–42 would have robbed the study of most of its statistical power to test cross-level interactions. Furthermore, it seemed likely that participants not providing any before-ratings differed from those providing before-ratings in a systematic way (having a different daily schedule). Therefore, the before ratings were not used in the main analyses reported below; instead, the after ratings were compared to the baseline. It should be acknowledged here that by excluding the “before” ratings, the chosen strategy cannot control for possible differences in outcomes between the baseline and intervention weeks due to factors other than the intervention, thus adding to the uncertainty of any conclusions made from the results.

For the modeling strategy, linear multilevel models regressing each outcome on the condition (baseline vs. intervention), personality traits, and their interaction, including a random intercept of the participants, were used. Baseline was used as the reference condition. The models were conducted separately for agreeableness and conscientiousness, first predicting the outcomes from baseline vs. agreeableness week (level 1 predictor), trait Agreeableness (level 2 predictor), and their interaction (cross-level interaction), and then predicting the outcomes from baseline vs. conscientiousness week, trait Conscientiousness, and their interaction. Equation 1 presents the model in mathematical terms.


$$\begin{array}{l} \begin{array}{c} y_{i}\sim N\left(\alpha_{j\left[i\right]}+\beta_{1}\left(cond_{after}\right),\sigma^{2}\right)\\ \alpha_{j}\sim N\left(\gamma_{0}^{\alpha }+\gamma_{1}^{\alpha }\left(Trait\right)+\gamma_{2}^{\alpha }\left(Trait\times cond_{after}\right),\sigma_{\alpha_{j}}^{2}\right),\;\\ for\;id\;j\;=\;1,\ldots ,J \end{array} \end{array}$$ (1)

*y*_*i*_ *refers to the level 1 outcome*, α_*j*[*i*]_ *is the level 1 intercept*, β_1_ *is the level 1 fixed effect of condition*, $\gamma_{0}^{\alpha }$ *is the participant intercept*, $\gamma_{1}^{\alpha }$ *is the personality trait fixed slope*, $\gamma_{2}^{\alpha }$ *is the cross-level interaction slope*, σ^2^*is the level 1 residual, and* $\sigma_{\alpha_{j}}^{2}$ *is the level 2 residual*.

### 2.6 Power considerations

The study was conducted without preregistration, and the sample recruited was a convenience sample. Therefore, power calculations were not performed prior to data collection. Indicative power calculations were, however, conducted prior to data analyses via a simulation approach using the R package *simr* (Green and MacLeod, 2016). First, the power for the condition effect (i.e., power for detecting a difference in a level 1 outcome between the two conditions) was investigated. According to the simulation results, with 70 participants (the lowest level 2 *N* used in analyses, as explained in the previous section), 15 observations per participant from baseline, and four from the “after” condition (the average numbers of observations per participant from both conditions; see next section), the dataset had 99% power to detect a mean difference between two conditions corresponding to a Cohen's *d* of 0.20 (i.e., a small effect) with a 0.05 alpha level.

Second, the power to detect a condition × trait interaction effect was simulated. According to the simulation results, the dataset had about 84% power to detect a condition × trait interaction effect with a size of ~0.20, i.e., a 0.20 unit difference in trait slope coefficients between two conditions, corresponding to, for instance, a slope of 0.10 in one condition and 0.30 in another. Changing effect sizes showed that the dataset had approximately 60% power of detecting a slope difference of 0.15, and >99% power of detecting a slope difference of 0.30. Thus, though the dataset was small, it was reasonably powered to detect a slope difference of approximately 0.20 or higher. However, the dataset was not adequately powered to detect a slope difference of 0.15 or smaller. The power calculations are available in the Online Appendix S1.

## 3 Results

### 3.1 Overview

Participants provided, in total, 1,072 observations from the baseline week (91% of the potential maximum of 1,184), 14.5 observations per participant on average (median = 15, range = 8–16). Participants provided a total of 256 “after” condition observations from the agreeableness week, four observations per participant on average (range = 0–4), and 259 “after” condition observations from the conscientiousness week, again four observations per participant on average (range = 0–5). Descriptive statistics of all the studied variables are presented in Table 2, and the intercorrelations between the variables are presented in Supplementary Tables S1–S4 in the online Appendix S1. Trait Agreeableness and trait Conscientiousness had a correlation of *r* = 0.19 (*p* = 0.070), trait Agreeableness had a correlation of *r* = 0.18 (*p* = 0.150) with ESM average agreeableness (across weeks), and trait Conscientiousness had a correlation of *r* = 0.44 (*p* < 0.001) with average state conscientiousness (across weeks). Between- and within-person correlations between ESM variables for each of the three weeks are presented in Supplementary Tables S1–S3 in the online Appendix S1, and correlations of Trait Agreeableness and Trait Conscientiousness with ESM averages from different weeks are presented in Supplementary Table S4.

**Table 2:** Means (standard deviations) of the studied variables (*N* = 74).

**Measure**	**Baseline week**	**Agreeable—week 1**	**Conscientious—week 2**
Positive affect	3.10 (0.88)	3.24 (0.87)	3.20 (0.81)
Negative affect	1.61 (0.70)	1.53 (0.64)	1.57 (0.61)
Stress	2.08 (1.00)	2.04 (0.99)	2.15 (1.01)
Tiredness	2.48 (1.07)	2.40 (0.99)	2.42 (1.01)
Agreeableness	3.44 (0.73)	3.61 (0.68)	3.60 (0.73)
Conscientiousness	3.57 (0.70)	3.69 (0.74)	3.84 (0.73)
Sociability	2.80 (1.18)	3.05 (1.13)	2.95 (1.18)
Insecurity	1.96 (1.00)	2.00 (0.95)	2.00 (0.94)
*n*_obs_	1,066–1,072	255–256	259
Trait Agreeableness	3.78 (0.50)		
Trait Conscientious	3.59 (0.71)		

All variables were measured on a scale from 1 to 5. Ratings from “before” conditions (N = 43, n = 140 for week 1 and N = 35, n = 94 for week 2) are not included. The number of observations varies for the baseline week and agreeableness week because some reports from those weeks were partial (only complete reports were obtained from the conscientiousness week).

### 3.2 Manipulation checks

All analyses were conducted using R statistical software (R Core Team, 2023). First, manipulation checks were conducted to determine whether the prompt affected self-rated agreeable and conscientious behavior in the intended way by conducting multilevel models with condition (baseline vs. before vs. after completing the prompt) predicting self-reported agreeable (week 1) and conscientious (week 2) behavior, including a random intercept of participants, using the lme4 package (Bates et al., 2015) function *lmer* in R.

Participants reported the highest agreeableness in the agreeableness week after completing the prompt (*M* = 3.60, *SE* = 0.06), the second highest in the baseline week (*M* = 3.43, *SE* = 0.06), and the lowest in the agreeableness week before the prompt (*M* = 3.08, *SE* = 0.07); all comparisons were significant at the *p* < 0.001 level. Differences in conscientiousness in the agreeableness week were smaller, *M* (*SE*) = 3.56 (0.05), 3.59 (0.07), and 3.67 (0.06) for the baseline, before, and after, respectively, although the difference between baseline and after-the-prompt ratings was significant at *p* = 0.038. Regarding conscientiousness, participants reported the highest conscientiousness after completing the prompt (*M* = 3.82, *SE* = 0.06), followed by baseline (*M* = 3.57, *SE* = 0.05), and before completing the prompt (*M* = 3.36, *SE* = 0.08). All differences were significant at *p* < 0.01 or lower. Furthermore, participants also reported higher agreeableness after the prompt (*M* = 3.58, *SE* = 0.06) than before the prompt (*M* = 3.35, *SE* = 0.08) or in the baseline week (*M* = 3.43, *SE* = 0.06), with both comparisons to the after-the-prompt ratings being significant (*p*s = 0.003 and < 0.001).

To check the divergent validity of the interventions, multilevel models regressing (1) momentary sociability and (2) momentary insecurity on the condition (baseline week vs. intervention week, after the prompt) were conducted separately for agreeableness and conscientiousness weeks. The only significant result was that sociability was higher in the agreeableness week after the prompt than at baseline week (*M*s 3.05 vs. 2.80, *p* = 0.001). However, the effect of condition on state agreeableness remained significant when adding (person-mean-centered) sociability as a predictor into the manipulation check model for agreeableness, although the effect of intervention on state agreeableness was smaller in the latter model (standardized effect sizes for condition were 0.12 and 0.07, *p*-values < 0.0001 and 0.004, respectively).

### 3.3 Main analyses

Multilevel models regressing the key dependent variables—positive affect, negative affect, stress, and tiredness—on condition (baseline week vs. after-the-prompt), corresponding trait, and condition trait interaction were conducted separately for agreeableness and conscientiousness weeks. The critical *p*-values—those related to the condition trait interactions—were subjected to Benjamini–Hochberg adjustment (BH; Benjamini and Hochberg, 1995) due to multiple testing. The BH adjustment was chosen because this study was exploratory and meant to be hypothesis-generating rather than confirmatory and because the clearest alternative, the Bonferroni correction, is generally considered rather stringent by contemporary standards, especially if the outcomes are related (Schulz and Grimes, 2005), as in this study. The equation for the model is shown in Equation 1.

**Figure 1:**
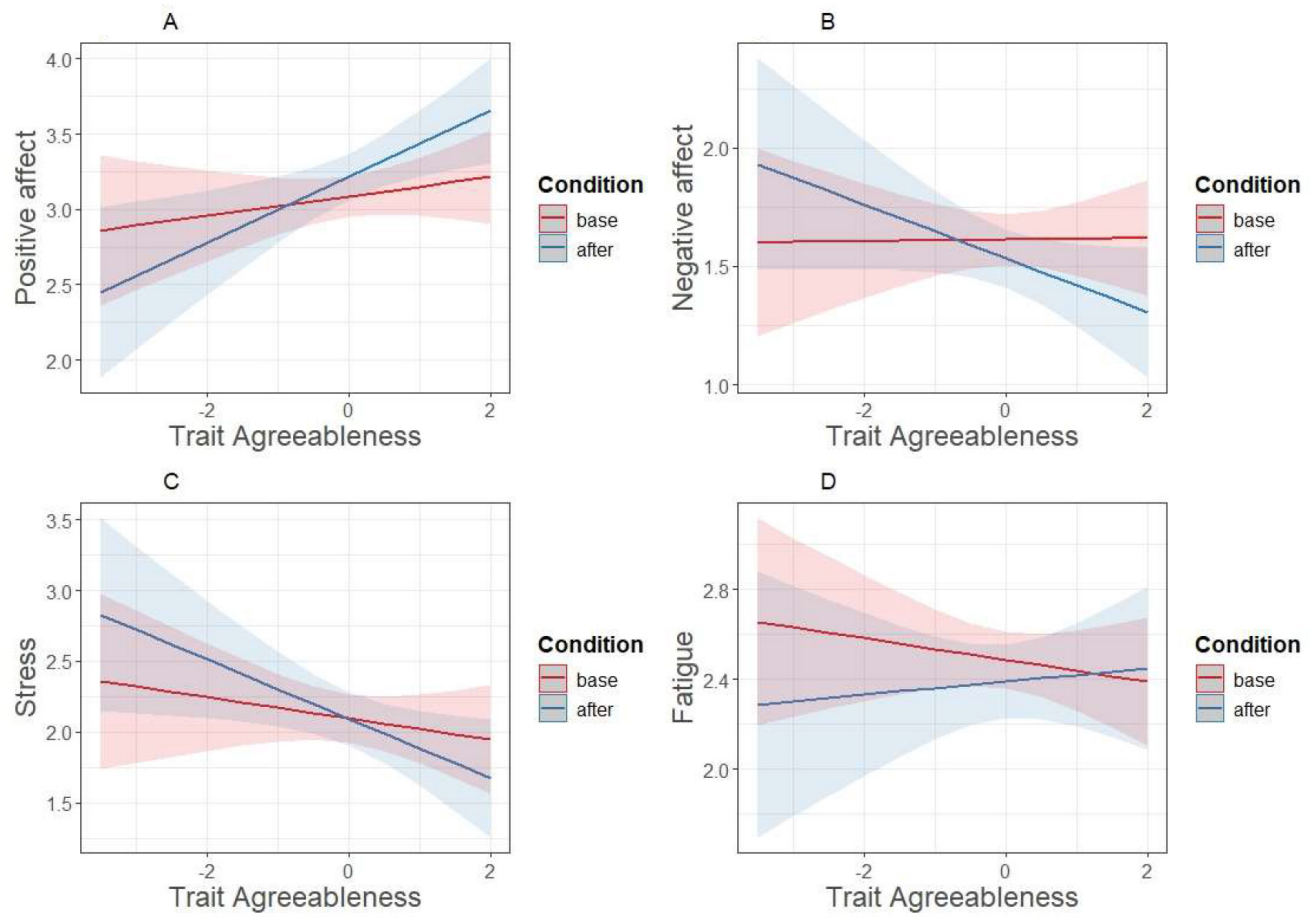
Trait Agreeableness × Condition interaction predicting outcomes in the agreeableness week. Base = baseline week; after = agreeableness week - after the prompt.

The results for agreeableness and conscientiousness are presented in Tables 3, 4. As shown in Table 3, the condition × trait Agreeableness interaction was significant for positive affect, negative affect, and stress after *p*-value adjustment. The interaction effects for agreeableness are plotted in Figure 1, and they show that the trait Agreeableness was more strongly related to positive affect, negative affect, and stress in the intervention condition than at baseline, predicting positive affect more positively and negative affect and stress more negatively in the intervention condition than at baseline. Simple slopes analyses (conducted via the emmeans package (Lenth, 2021) using the *emtrends* function) showed that trait Agreeableness was related to positive affect in the intervention condition, *B* = 0.21, *SE* = 0.08 (95% CIs = 0.05, 0.36), but not in the baseline condition, *B* = 0.07, *SE* = 0.07 (95% CIs = −0.07, 0.21). Furthermore, trait Agreeableness was negatively related to momentary stress in the intervention condition, *B* = −0.20, *SE* = 0.09 (95% CIs = −0.39; −0.02), but not in the baseline condition, *B* = −0.08, *SE* = 0.09 (95% CIs = −0.25, 0.10). For negative affect, neither of the simple slopes was significantly different from zero (intervention *B* = −0.10, *SE* = 0.06, CIs −0.22; 0.03; baseline *B* = 0.01, *SE* = 0.05, CIs −0.10; 0.11).

**Table 3:** Results of multilevel models with condition (baseline vs. intervention), Trait Agreeableness, and their interaction predicting the momentary wellbeing outcomes (*N* = 74).

**Parameter**	**Positive affect**	***p-* value**	**Negative affect**	***p-* value**	**Stress**	***p-* value**	**Tiredness**	***p-* value**
**Fixed effects**								
Intercept	3.09		1.61		2.10		2.49	
Cond_after_	0.12 (0.05)	0.012	−0.07 (0.04)	0.071	−0.00 (0.05)	0.993	−0.08 (0.07)	0.206
Trait A	0.07 (0.07)	0.321	0.01 (0.05)	0.922	−0.08 (0.09)	0.382	−0.04 (0.06)	0.485
Cond_after_ × Trait A	0.14 (0.05)	0.022	−0.10 (0.04)	0.022	−0.13 (0.05)	0.024	0.09 (0.07)	0.242
**Random effects**								
τ_00_ (participant)	0.31		0.20		0.50		0.24	
Residual	0.45		0.28		0.50		0.87	
ICC	0.41		0.42		0.50		0.21	

Fixed effects are unstandardized estimates (SEs) from multilevel models. Baseline was the reference condition. τ_00_ = participant-related variance in outcome. Adjusted ICCs are presented. Cond = Condition (baseline vs. after the prompt). A = Agreeableness. p-values of the interaction effects are adjusted using the Benjamini–Hochberg method.

**Table 4:** Results of multilevel models with condition (baseline vs. after), trait Conscientiousness, and their interaction predicting the momentary wellbeing outcomes.

**Parameter**	**Positive affect**	***p-*value**	**Negative affect**	***p-* value**	**Stress**	***p-*value**	**Tiredness**	***p-* value**
**Fixed effects**								
Intercept	3.09		1.61		2.09		2.49	
Cond_after_	0.08 (0.05)	0.100	0.00 (0.04)	0.925	0.11 (0.05)	0.035	−0.08 (0.07)	0.232
Trait C	0.09 (0.07)	0.189	−0.07 (0.05)	0.192	−0.08 (0.08)	0.329	0.02 (0.06)	0.783
Cond_after_ × Trait C	−0.03 (0.05)	0.576	−0.05 (0.04)	0.243	0.02 (0.05)	0.682	0.12 (0.07)	0.163
**Random effects**								
τ_00_ (participant)	0.30		0.18		0.48		0.21	
Residual	0.45		0.28		0.53		0.91	
ICC	0.39		0.39		0.47		0.19	

Fixed effects are unstandardized estimates (SEs) from multilevel models. Baseline was the reference condition. τ_00_ = participant-related variance in outcome. Adjusted ICCs are presented. Cond = Condition (baseline vs. after the prompt). C = Conscientiousness. p-values of the interaction effects are adjusted using the Benjamini–Hochberg method.

**Figure 2:**
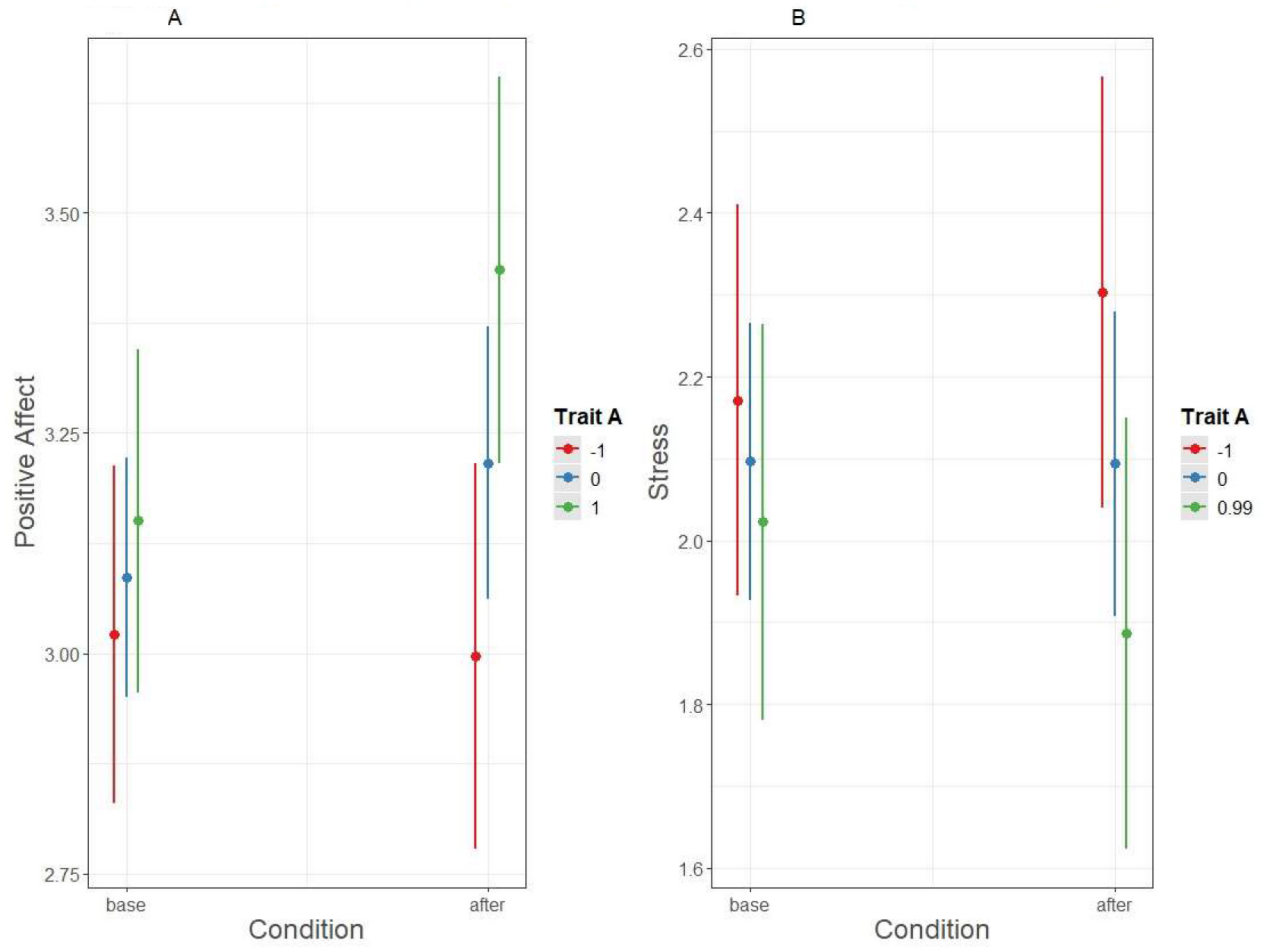
Positive affect and stress during baseline and intervention for participants at mean and at ± 1 *SD* of trait agreeableness.

To further explore two of the significant interaction results, the interactions for positive affect and stress were plotted as mean differences in the outcomes at the mean and ± 1 SD of the mean of trait Agreeableness. Figure 2 shows that participants with trait Agreeableness at 1 *SD* below the mean had, on average, about the same level of positive affect at baseline and during the intervention, but participants with trait Agreeableness at the mean or 1 *SD* above the mean had higher positive affect during the intervention. Regarding stress, participants with a mean trait Agreeableness had about the same stress level at baseline and after the prompt. However, participants with trait Agreeableness 1 *SD* below the mean had higher stress after the prompt than at baseline, whereas participants with trait Agreeableness 1 *SD* above the mean had lower stress after the prompt than at baseline.

**Figure 3:**
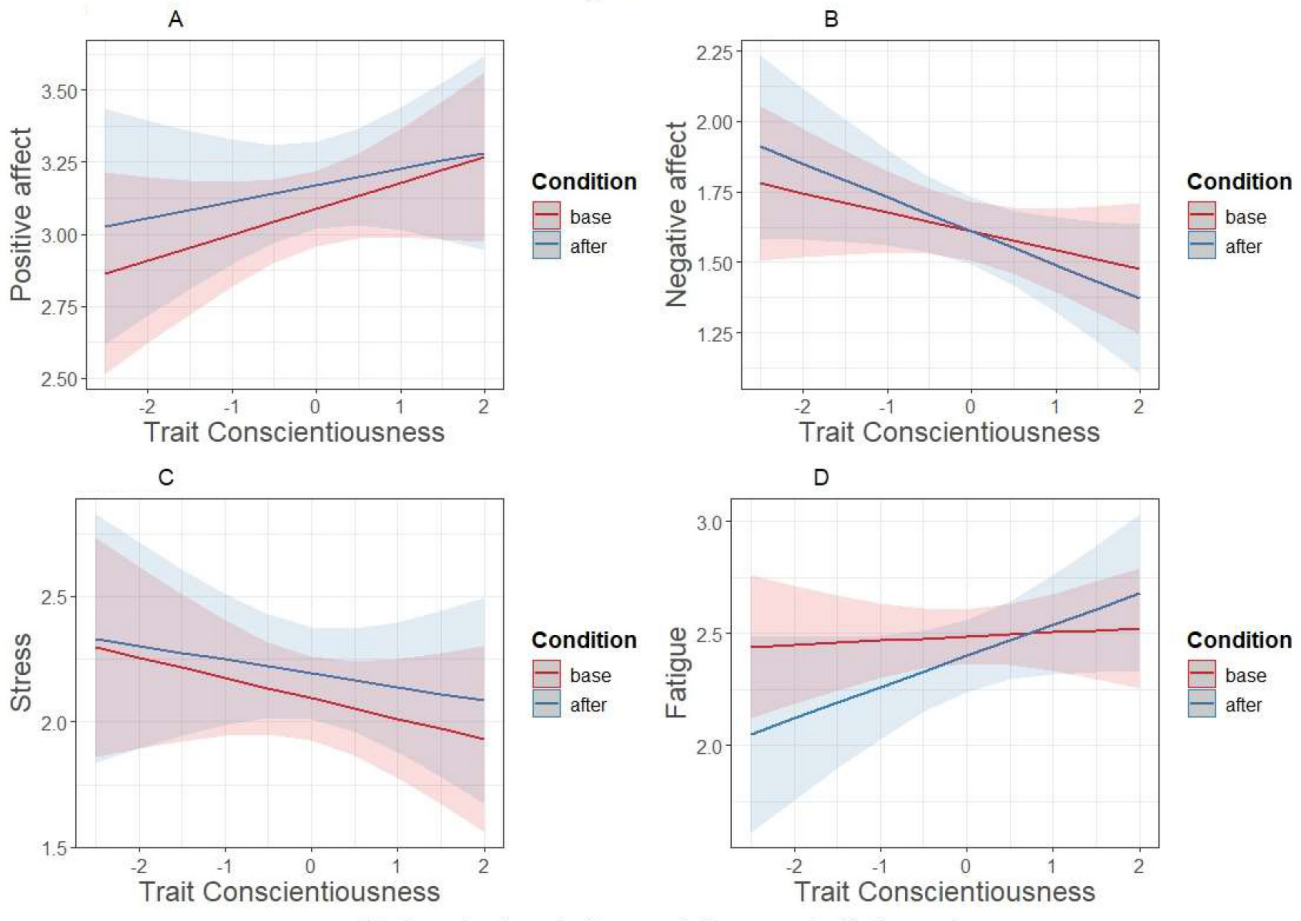
Trait Conscientiousness × condition interaction predicting outcomes in the agreeableness week. Base = baseline week; after = conscientiousness week - after the prompt.

By contrast, none of the interactions were significant for trait Conscientiousness (Figure 3); generally, condition was unrelated to outcomes in the conscientiousness week, and trait Conscientiousness was positively related to positive affect and negatively to negative outcomes regardless of condition.^1^

Next, we investigated whether traits moderated the effect of condition on behavior by regressing the behavioral variables on the corresponding trait, condition, and their interaction (these analyses are presented in Supplementary Tables S5, S6 in the Online Appendix S1). Indeed, trait Agreeableness moderated the effect of condition on behavioral agreeableness (interaction term estimate = 0.39, *SE* =0.09, *p* < 0.001 after the adjustment) so that trait Agreeableness was more strongly positively related to agreeable behavior in the after condition (Figure 2A); the baseline slope was 0.15 (95% CIs = −0.13, 0.44), and the after condition slope was 0.55 (95% CIs = 0.23, 0.86). In contrast, trait Conscientiousness did not moderate the effect of condition on conscientious behavior (interaction term *B* = −0.08, *SE* = 0.09, *p* = 0.523, Figure 2B); instead, condition and trait Conscientiousness had independent, positive relationship with conscientious behavior.

### 3.4 Control analyses

Due to the lack of before-prompt reports, the intervention prompt and dependent variables were not as well-aligned temporally as originally planned. That is, many after-the-prompt ratings of affect, stress, and fatigue could have occurred a relatively long time after the agreeable or conscientious act was conducted. This undermines the internal validity of the design. To deal with this problem, reports regarding the time that had passed after conducting the act were investigated. In the agreeableness week, 83 reports were completed less than 1 h after the act, 98 after 1–2 h, 39 after 2–3 h, and 31 after 3+ h. In the conscientiousness week, the corresponding frequencies were 83, 96, 48, and 30, respectively. Thus, the majority of after-act reports were completed within 2 h of the act.

To investigate the role of time passed after the act in the results, the models predicting momentary affect, stress, and fatigue were re-run (1) including the time variable as a covariate and (2) including only those after-the-act reports that were provided 2 h or more after the act. The condition × Agreeableness interaction effect remained significant when the time variable was included as a covariate for positive affect (*B* = 0.12, *SE* = 0.05, *p* = 0.010), negative affect (*B* = −0.09, *SE* = 0.04, *p* = 0.018), and stress (*B* = −0.13, *SE* = 0.05, *p* = 0.008). Furthermore, the condition × Agreeableness interaction effect remained significant in the model including only late (2+ h after) reports for positive affect (*B* = 0.18, *SE* = 0.08, *p* = 0.027), negative affect (*B* = −0.12, *SE* = 0.06, *p* = 0.049), and stress (*B* = −0.23, *SE* = 0.08, *p* = 0.005). The corresponding effects from the Conscientiousness week remained non-significant (*B*s < |0.14|, *p*s > 0.052). The agreeableness week interaction effect on fatigue approached significance when controlling for time (*B* = 0.13, *SE* = 0.07, *p* = 0.05), but not when only later reports were included (*B* = 0.10, *SE* = 0.11, *p* = 0.384).

Next, models using the sparse available before-and-after data were conducted on positive affect and stress. The interaction effect between condition and trait Agreeableness on positive affect was significant, *B* = 0.15, *SE* = 0.05, *p* = 0.001, and it was visually similar to the one presented in Figure 1A. The effect on stress was also significant (*B* = −0.14, *SE* = 0.05, *p* = 0.006), and it was visually similar to that found in the baseline-after stress model (Figure 1C).

Thus, the control analyses suggested that the intervention effect was evident even in later reports and could be captured even in the absence of prior reports. Furthermore, the interaction effects of condition and Agreeableness on positive affect and stress in the before-and-after data closely resembled the effect found in the baseline-after data. Details of the control analyses are available in the Online Appendix S1.

## 4 Discussion

### 4.1 General discussion

The idea that behaving in ways that are incompatible with one's personality might undermine wellbeing or lead to mental depletion has gained a considerable amount of interest within personality science, but results so far have mostly not supported this idea [Fleeson et al., 2002; Kuijpers et al., 2024; Leikas et al., 2021; Margolis and Lyubomirsky, 2020; van Allen et al., 2021; Zelenski et al., 2012, but see (Côté and Moskowitz 1998), (Jacques-Hamilton et al. 2019) and (Kuijpers et al. 2022) for exceptions]. The results of the present study, among the first to investigate agreeable and conscientious behavior in an intervention framework, suggest that trait Agreeableness may moderate the relationships between induced agreeable behavior and feelings. In particular, the results support the idea that there may be psychological costs of induced counterdispositional behavior (and psychological rewards of induced trait-consistent behavior) in the domain of Agreeableness. In contrast, trait Conscientiousness did not moderate the relationship between induced conscientious behavior and feelings. The results are discussed below in turn.

It is well-established that trait Agreeableness is positively related to higher average levels of agreeable behavior in everyday life (Fleeson and Gallagher, 2009). Broadly in line with this intuitively understandable finding, the present results suggest that induced agreeable behavior might be less stressful and more affectively rewarding for individuals with higher trait Agreeableness, as compared to individuals with lower trait Agreeableness. Furthermore, the results suggested affective costs of induced agreeableness to those with low trait Agreeableness, because those with low trait Agreeableness reported lower positive affect after induced agreeableness than at baseline, whereas the opposite was true for those with high trait Agreeableness (Figures 1A, 2A). Furthermore, low-Agreeableness individuals also reported higher stress and higher negative affect after being induced to behave agreeably than at baseline, whereas the opposite was true for high-Agreeableness individuals.

In previous studies on state-trait interaction effects in the domain of Agreeableness, (Leikas et al. 2021) found no interaction effects on momentary affect, fatigue, or self-control, but (Côté and Moskowitz 1998) found an interaction effect for positive and negative affect in an event-contingent ESM design. Thus, previous research on this topic has been inconclusive (and sparse). However, considering previous research together with the current results, it seems possible that trait Agreeableness does not moderate agreeable behavior-outcome relationships when agreeable behavior occurs naturally but may moderate them when agreeable behavior is enacted via intervention (or, possibly, when measured in an event-contingent manner).

The present study could not investigate the exact reasons for this disparity, but it should be noted that the intervention procedure used was likely to induce controlled (as opposed to autonomous) motivation for the agreeable behavior in the intervention week.^2^ Self-determination theory (Ryan and Deci, 2006) suggests that doing things based on controlled motivation (i.e., because of external demands or rewards) is related to worse performance and psychological costs as compared to doing things based on autonomous motivation (i.e., out of one's own interest and volition), and there is some evidence for this (Tadić Vujčić et al., 2017). Thus, participants acting agreeably for external reasons may have played a role. This idea is also broadly compatible with the emotional labor literature showing that customer service workers often experience stress and negative affect after having to “fake” friendliness in work situations (Hülsheger and Schewe, 2011). It seems possible that the participants in the present study were engaging in emotional work when completing the agreeableness prompts, and this may have been more depleting for participants with low trait Agreeableness.

Several theories on why counter-trait behavior might come with psychological costs have been presented in the literature, such as cognitive dissonance evoked by counter-trait behavior (Festinger, 1957) and trait-consistent behaviors being more automated and habitual (Wood et al., 2002), leading to trait-inconsistent behaviors requiring more self-regulatory effort and thus consuming more psychological resources (see Gallagher et al., 2011). Furthermore, a reward-sensitivity explanation has been proposed, especially in the context of extraversion (Smillie et al., 2012), which suggests that those with a high level of a given trait experience trait-related behavior as more rewarding. The present study did not test these explicit mechanisms but provided some evidence compatible with all of them in the domain of Agreeableness. Nevertheless, it should be acknowledged that the effect sizes here were modest, and based on them, trait moderation is not likely to play a huge role in the everyday life dynamic between agreeable behavior and feelings.

As for the domain of Conscientiousness, no interaction effects were found. Rather, both trait Conscientiousness and induced conscientious behavior were independently related to better mood and lower stress. The results are in line with previous observational studies that have shown that behaving more conscientiously than typically is generally related to better mood and being less tired, with no interactions by trait Conscientiousness or average level of conscientious behavior (Kritzler et al., 2020; Kuijpers et al., 2024; Leikas et al., 2021). The present results show that a similar relationship exists between induced conscientious behavior and feelings. Furthermore, the current results are also in line with previous research showing a (small) positive relationship between trait Conscientiousness and momentary and daily positive affect (Howell et al., 2017; Kritzler et al., 2020).

Conscientious behavior is, by its nature, often resource-consuming—after all, it literally means acting responsibly, dutifully, productively, orderly, and/or in a goal-directed manner. Therefore, whether such behavior is easier for people with high trait Conscientiousness and more difficult for people with low trait Conscientiousness is an intriguing question. The present results add to the accumulating literature suggesting that this is not so, but that conscientious behavior is in fact related to higher momentary wellbeing for everyone, regardless of personality traits or habitual levels of behavior (Kuijpers et al., 2024; Leikas et al., 2021).

Why does the trait Conscientiousness not moderate the state conscientiousness–outcome relationships if the trait Agreeableness moderates the corresponding relationships? The latter result needs to be replicated in future studies before it can be considered reliable. However, it is possible that conscientious and agreeable behaviors and their relationships with their corresponding traits are, in some ways, different. One difference might be that conscientious behavior, while typically effortful and resource-consuming, often leads to external rewards (see text footnote ^2^) (e.g., good grades, positive feedback at work) and/or desirable tangible outcomes for oneself (e.g., a clean home, feelings of accomplishment). While agreeable behavior is likely to have positive consequences, such as pleasant everyday life interactions, and well-functioning social relationships, to the actor, it may be that agreeable behavior is not tied to concrete rewards to the same extent as conscientious behavior. If future studies replicate the Agreeableness-related findings obtained here, this is a possibility worth considering and investigating further.

### 4.2 Limitations

The present study had several limitations, the most important being the small sample size, the high proportion of women in the sample, and the lack of pre-registration. Additionally, the sample consisted of students who typically had relatively high autonomy in planning their daily schedules. Therefore, participants may have been able to respond to the prompts more easily than adults in typical full-time jobs. The results are therefore not as reliable and generalizable as they could be and must be considered in this light. The within-subject, intensive-longitudinal design counteracted these caveats to an extent, as well as power calculations, suggesting that the dataset was reasonably powered to detect the effects of interest. Nevertheless, the results should be treated as preliminary and should not be generalized to the population of mostly female Western university students.

Other limitations include issues related to the intervention procedure. The study design did not implement stringent experimental controls; therefore, causal conclusions could not be drawn from the results. Furthermore, comparisons had to be conducted between the baseline week and after-the-prompt observations, and the before-prompt scores from the intervention weeks could not be used. There may have been uncontrolled differences between the baseline and intervention weeks, which may have affected the results.

Additionally, the intervention procedure was susceptible to social desirability concerns. In particular, participants may have felt a need to report having completed the prompt, even if they did not. Unfortunately, there was no in-built control in the design for this possibility; therefore, it is possible that such false-positive reports undermined the validity of the results. However, this is perhaps somewhat unlikely to be a huge problem, given that most participants reported having completed the prompt at the first measurement occasion of the day on both intervention weeks. It would seem that the need to lie about completing the prompt because of reputational concerns would only arise if the participant had not completed the prompt by the last measurement of the day.

As is common in daily life studies, the intervention, as well as the momentary measures, could only cover the constructs of interest partially. In terms of the Big Five model (Goldberg, 1992), the agreeableness prompt focused on the kindness/warmth aspect of Agreeableness (and in terms of the Five-Factor model, on the facet Altruism), and the results are thus uninformative with regard to the other aspects/facets of Agreeableness, such as trust, compliance, and modesty. This emphasis also makes it difficult to interpret the results as being unique to the effects of induced agreeableness as separate from the effects of prosocial behavior interventions, which usually have shown similar effects on affective wellbeing (Byrne et al., 2023; Nelson et al., 2015, although prosocial intervention prompts typically have a more narrow/concrete scope than the ones used here). Similarly, Conscientiousness prompts focus on dependability and carefulness (dutifulness and order in FFM terms) and cannot speak of the effects of, say, induced neatness, efficiency, or achievement striving on the outcomes. Finally, the scales used to measure outcomes were also brief and narrow and thus did not cover the full range of positive and negative affective experiences (Watson et al., 1988).

Finally, traditionally measured personality traits were used as indicators of baseline disposition in this study. Another way of capturing habitual tendencies of behavior is to use a behavioral summary—most commonly the average—derived from the ESM phase. ESM-based behavioral averages tend to correlate substantially (*r*s = 0.35–0.50) with corresponding traits (Fleeson and Gallagher, 2009). Such averages have the advantage of literally representing individual differences in behavioral tendencies over some time period, and previous studies have found counterdispositional cost effects using such averages (Kuijpers et al., 2022). In the present study, the use of such averages was not deemed feasible because of the short baseline period; however, it should be acknowledged that the results could have been different if reliable behavioral averages had been used instead of trait measures. The theoretical and conceptual relationship between traditionally measured traits and empirical behavioral averages is an important contemporary question in experience-sampling research on personality.

## 5 Conclusion

The interplay between personality, behavior, and important outcome variables is a lasting theme in social and personality psychology. A topic evoking particular interest has been the role of traits in modifying behavior–outcome relations. The majority of the studies have suggested that personality traits do not play a role in these relationships. The present study was in line with these results with regard to the domain of Conscientiousness. However, it also suggested—to my knowledge, the first time in the field—that trait Agreeableness may affect agreeable behavior–outcome relations by making it slightly easier or more rewarding for those with high trait Agreeableness to behave in an agreeable way. If this result turns out to be reliable in future replications, it has the potential to further our understanding of the nature of the trait Agreeableness and agreeable behavior. Together, the results also suggest that traits identified by the major personality models may differ from each other in important ways in terms of everyday life dynamics.

## Data Availability

The original contributions presented in the study are publicly available. This data can be found here: https://osf.io/g6vbu/.

## Footnotes

1 For tiredness, there was a visually discernible tendency toward trait Conscientiousness being positively related to tiredness in the after condition and unrelated to tiredness in the baseline condition. To explore this pattern further, simple slopes were extracted from the model, and their confidence intervals were computed. The trait Conscientiousness was more strongly related to tiredness in the after condition, *B* = 0.21 (*SE* = 0.12, 95 % CIs −0.02; 0.44), than in the baseline condition, *B* = 0.02 (*SE* = 0.09, CIs −0.15, 0.20), though the difference was, as noted, not statistically significant.

2 I thank an anonymous reviewer for this point.
